# A Comprehensive Molecular and Clinical Study of Patients with Young-Onset Colorectal Cancer

**DOI:** 10.3390/cancers17172763

**Published:** 2025-08-25

**Authors:** Elham Nasrollahi, Shuaichao Wang, Rami Yanes, Cyndi Gonzalez Gomez, Tara Magge, Abigail Overacre, Ronan Hsieh, Ashley Mcfarquhar, Curtis Tatsuoka, Aatur Singhi, Anwaar Saeed, Ibrahim Halil Sahin

**Affiliations:** 1 Department of Internal Medicine, University of Pittsburgh Medical Center, Pittsburgh, PA15213, USAshw252@pitt.edu (S.W.); maggetl@upmc.edu (T.M.); saeeda3@upmc.edu (A.S.); 2 University of Pittsburgh Medical Center (UPMC), Harrisburg, PA 17101, USA; 3 UPMC Hillman Cancer Center, Pittsburgh, PA 15232, USA; 4 University of Pittsburgh Medical Center (UPMC), Pittsburgh, PA 15232, USA; overacre@pitt.edu; 5 Department of Immunology, School of Medicine, University of Pittsburgh, Pittsburgh, PA 15213, USA; 6 Fred Hutchinson Cancer Center, University of Washington, Seattle, WA 98109, USA; ronan.hsieh@providence.org; 7 Department of Medicine, School of Medicine, University of Washington, Seattle, WA 98105, USA

**Keywords:** early-onset colorectal cancer, young-onset CRC, molecular profiling, KRAS, BRAF, microsatellite instability, POLE, survival, next-generation sequencing, prognostic biomarkers

## Abstract

**Background**: Young-onset colorectal cancer (YO-CRC) has emerged as a distinct clinical entity, often presenting at advanced stages. Despite the increasing incidence, the molecular and clinical underpinnings of YO-CRC remain underexplored. This study aims to characterize the clinical and molecular features of YO-CRC and to evaluate their impact on OS. **Methods**: We reviewed 110 patients diagnosed with YO-CRC at our institution who underwent next-generation sequencing. Demographic, clinical, and molecular data, including age, gender, race, tumor location, cancer stage, and mutation status (KRAS, NRAS, BRAF, POLE, ERBB-2/HER2, microsatellite status), were collected by reviewing electronic medical records. For OS analysis, we focused on patients diagnosed with de novo stage IV. Cox proportional hazards regression and Kaplan–Meier survival analysis were utilized to assess the association of these factors with OS, with statistical significance determined by a *p*-value threshold of <0.05. **Results**: Among 110 patients, *n* = 44 (40%) presented with local disease (stage 1–3), while *n* = 66 (60%) presented with de novo metastatic disease at the time of diagnosis. The median age at diagnosis was 44.5 years. The cohort consisted of 64% males and 36% females, with 84% of patients identified as White. Most tumors were left-sided (77%), including the distal colon/sigmoid (44%) and rectum (33%). KRAS and BRAF mutations were present in 36% and 5.5%, respectively. ERBB-2/HER2 amplification and microsatellite instability were observed in 4.5% and 6.4%, respectively. Tumor mutation burden (TMB) was <10 in 57% of patients, with 14% having TMB > 20. CNV analysis revealed that 14% of patients had copy gains, 12% had concurrent gains/losses, and 31% had copy losses. Among 66 patients with de novo metastatic disease, 44% had died by the time of analysis, with a median overall survival (OS) of 43.6 months (95% CI, 28.7—not reached). KRAS mutations were found to be significantly associated with worse survival outcomes. Cox regression analysis reveals the prognostic significance of KRAS status, with a hazard ratio (HR) of 3.52 (95% CI: 1.59–7.76, *p* = 0.002), indicating a significantly higher risk of death for KRAS-mutant YO-CRC patients. **Conclusions**: Patients with YO-CRC are more likely to present with de novo metastatic disease and left-sided tumors with distinct molecular characteristics. KRAS mutations are a key prognostic factor in YO-CRC, highlighting the need for therapeutic interventions to improve outcomes in this high-risk group.

## Simple Summary

Young-onset colorectal cancer (YO-CRC), defined as CRC diagnosed before age 50, is increasing in incidence and often presents with advanced disease. This study was conducted to provide comprehensive clinical and molecular characteristics in a cohort of 110 patients with YO-CRC using the institutional molecular and clinical database. We found that most patients had de novo stage IV disease and had left-sided tumors harboring predominantly exon-2 KRAS mutations (87%), while the overall rate of KRAS mutations was relatively lower (36%) than the known incidence of KRAS mutations in the overall population. Among patients presented with metastatic disease, the KRAS mutation was associated with significantly shorter survival, revealing its prognostic role among those with YO-CRC. These findings underscore the importance of molecular profiling and its potential role in guiding personalized treatment strategies for younger CRC patients.

## 1. Introduction

Colorectal cancer (CRC) is the third most commonly diagnosed cancer in the United States and the second leading cause of cancer-related death [1]. While the overall incidence of CRC has declined among older adults due to increased screening, the incidence of young-onset colorectal cancer (YO-CRC), defined as CRC diagnosed before age 50, has been rising steadily [2]. In the United States, the proportion of CRC cases occurring in individuals under 55 years has nearly doubled since 1995, and by 2030, the incidence of CRC is projected to increase by 28–46% among individuals aged 35–49 and by 90–124% among those aged 20–34 [3,4].

Risk factors for YO-CRC are not well defined, and several risk factors that are linked with regular-onset colorectal cancer were also associated with YO-CRC [1]. These risk factors include sedentary lifestyle, excessive sugar and alcoholic beverage consumption, increased body mass index and serum triglyceride levels, Western diet, increased red meat consumption, and smoking [5,6,7,8]. Gut microbiome, which is associated with increased inflammation leading to carcinogenesis, was also identified to be linked to YO-CRC in cohort studies [9]. It is, however, unclear why these traditional risk factors trigger carcinogenesis early in some individuals, and further research is warranted to better understand whether intrauterine and childhood exposures have any role in the early trigger of carcinogenesis and its association with YO-CRC in Western countries [10].

Patients with YO-CRC are more likely to present with left-sided tumors, high-grade histology, and advanced-stage disease at diagnosis [11,12]. Pathogenic germline variants are identified in 16–20% of cases, most commonly involving mismatch repair genes (*MLH1*, *MSH2*, *MSH6*, *PMS2*), *APC*, and *MUTYH* [13]. Somatic alterations in YO-CRC differ from those observed in older adults: *BRAF V600E* mutations occur in <5% of cases; *KRAS* mutations are found in 40–50%, primarily in exfon 2; microsatellite instability (MSI) is present in 15–20%; *POLE* or *POLD1* mutations are reported in 3–5%; and *HER2* amplification is observed in 2–3% [11,14,15,16]. However, it is important to note that various studies reported highly different rates of KRAS mutations among patients with YO-CRC. In a landmark study, the investigators reported increased rates of KRAS mutations among patients with young-onset CRC [13]. Notably, another study suggested the lower rates of KRAS mutations, indicating there is still a significant unmet need for research to better define molecular and clinical characteristics of YO-CRC [15]

Limited data on the prognostic significance of these molecular alterations in YO-CRC are available. In this study, we describe the clinical characteristics and molecular profiles of a cohort of patients with YO-CRC, including the distribution of *KRAS*, *BRAF*, microsatellite instability (MSI), *HER2* amplification, and other alterations. We also assess the association between molecular features and tumor sidedness and evaluate survival outcomes.

## 2. Methods

### 2.1. Study Population and Data Collection

**Figure 1:**
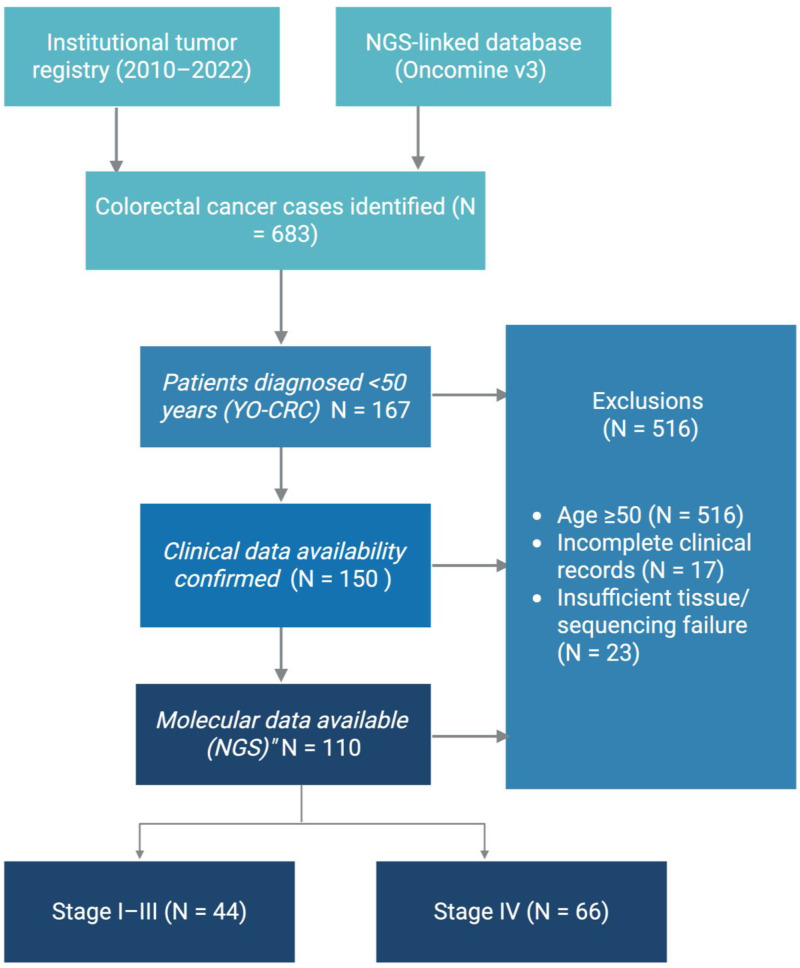
Flow diagram.

We performed a retrospective study of patients diagnosed with young-onset colorectal cancer (YO-CRC) at the University of Pittsburgh School of Medicine and University of Pittsburgh Medical Center, and its Network between 2010 and 2022. Eligible patients were those diagnosed before age 50 and had available next-generation sequencing (NGS) results from clinical genomic profiling (Figure 1). Clinical and molecular data were collected under the University of Pittsburgh IRB-approved protocol (STUDY20070085).

Demographic, clinical, and molecular variables were extracted from the electronic medical records and institutional tumor registry. Variables of interest included age at diagnosis, sex, race/ethnicity, tumor location, and stage at presentation. Tumor location was categorized as right-sided (cecum, ascending colon), transverse, or left-sided (descending colon, sigmoid, rectum). Staging was performed using AJCC 7th or 8th edition criteria by treating physicians.

Molecular data included alterations in KRAS, NRAS, BRAF, POLE, POLD1, ERBB2 (HER2), microsatellite instability (MSI) status, tumor mutational burden (TMB), and copy number variations (CNVs). KRAS mutations were subclassified by exon. HER2 amplification was determined from copy number data, and MSI status was categorized as MSI-high or microsatellite stable. TMB was calculated as the number of mutations per megabase (mut/Mb) and stratified into <10, 10–20, and >20.

### 2.2. Statistical Analysis

Descriptive statistics were used to summarize baseline demographic and clinical characteristics. Comparisons between groups were performed using the chi-square or Fisher’s exact test for categorical variables and the Mann–Whitney U test for continuous variables.

Primary survival analyses focused on individuals with de novo Stage IV disease. Kaplan–Meier curves were used to estimate OS, and the log-rank test was used for group comparisons. Multivariable Cox proportional hazards regression was used to evaluate associations between OS and clinical variables, including age, sex, and tumor sidedness. The proportional hazards assumption was assessed using Schoenfeld residuals. Hazard ratios (HRs), 95% confidence intervals (CIs), and two-sided *p*-values were reported.

### 2.3. Molecular Testing

We utilized in-house expanded targeted NGS-based testing from DNA and mRNA (which includes 161 cancer-relevant driver genes and 760 fusion genes), and the samples were analyzed in the MGP lab at UPMC using the Oncomine Comprehensive Assay v3 (Oncomine) DNA and RNA primer sets (Thermo Fisher Scientific; 168 Third Avenue, Waltham, MA 02451, USA) by using the manufacturer’s protocol. In principle, genomic material quantity and quality checks are routinely conducted using the 4200 TapeStation (Agilent Technologies, Santa Clara, CA, USA). The complementary DNA is created from mRNA by reverse transcription. Then the total DNA and reverse transcribed RNA are subjected to PCR to amplify the genomic regions of interest for testing. Massive parallel sequencing is performed by using an Ion GeneStudio S5 Prime System according to the manufacturer’s instructions (Thermo Fisher Scientific), and data are then examined with Variant Explorer (UPMC) for single-nucleotide variants, insertions, deletions, copy number alterations, and RNA fusion genes.

## 3. Results

### 3.1. Patient Characteristics

In total, 110 young-onset CRC patients were included in this analysis. The median age at diagnosis was 44 years (interquartile range [IQR] 40–47 years). There was a slight male predominance, with 51.8% of patients being male. Most patients presented with tumors in the left side of the colon: 63.6% had left-sided primaries, 28.2% had right-sided primaries, and 8.2% had transverse colon tumors (Table 1). At diagnosis, 60.0% of patients had synchronous metastatic disease (stage IV), whereas the remaining 40.0% presented with localized Stage I–III disease. Performance status was generally good in this cohort: among 91 patients with available ECOG performance data, 66 (72.5%) had an ECOG score of 0–1 (the remaining 27.5% having ECOG score ≥ 2).

### 3.2. Molecular Findings

Molecular profiling revealed that KRAS mutations were the most common oncogenic driver alteration, detected in 36.4% (40/110) of tumors. These KRAS mutations predominantly involved exon 2 (87%). This pattern was noted in both those with de novo metastatic disease (83%) and those who presented with earlier stage at the time of diagnosis (93%). BRAF mutations were identified in 5.5% (6/110) of patients, including four cases with the V600E variant (3.6%). Pathogenic polymerase proofreading gene mutations (POLE or POLD1) were found in 10.0% (11/110) of patients. ERBB2 (HER2) amplification was observed in 4.5% (5/110) of tumors. A microsatellite instability-high (MSI-H) phenotype was present in 6.4% (7/110) of cases, with the remainder being microsatellite stable (Table 2).

Tumor mutational burden (TMB) ≥ 10 mut/Mb was observed in 42.7% (47/110) of tumors, including approximately 14% (15/110) of patients with TMB > 20 mut/Mb. NTRK gene fusions were rare, identified in only 1.8% (2/110) of patients. Copy number variation analysis demonstrated that 14% (15/110) of tumors had focal copy number gains, 31% (34/110) had copy number losses, and an additional 12% (13/110) showed a combination of both gains and losses.

### 3.3. Survival Analysis

**Figure 2:**
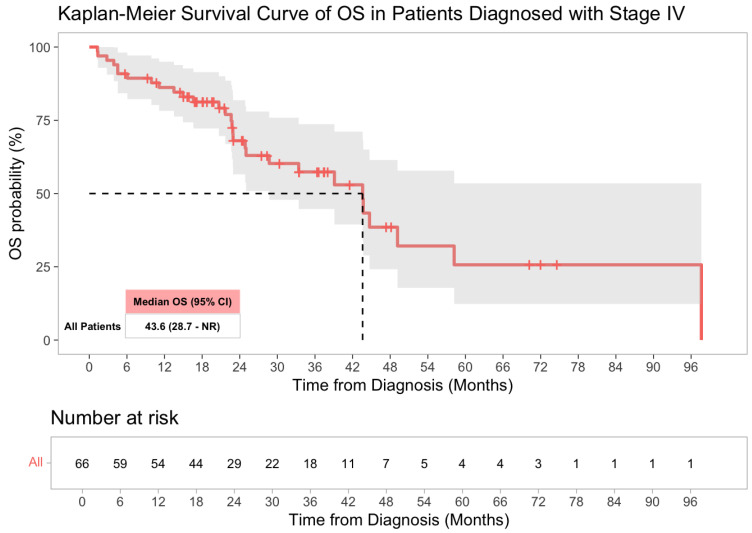
Kaplan–Meier overall survival curve for patients diagnosed with stage IV colorectal cancer.

**Figure 3:**
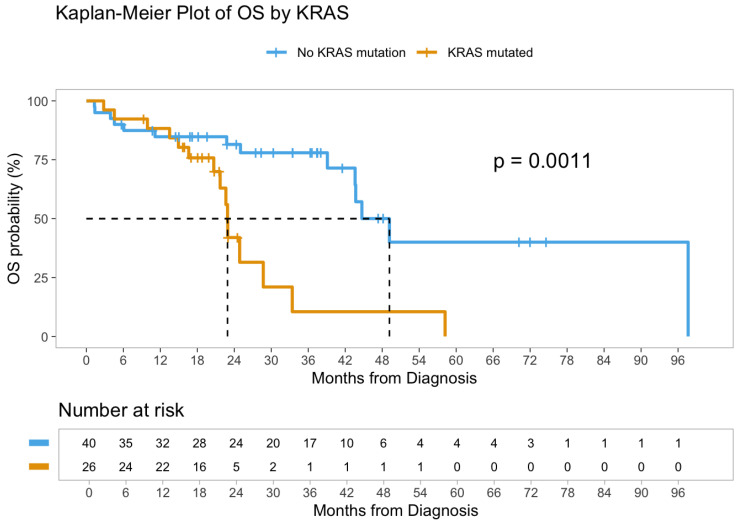
Kaplan–Meier plot of overall survival by KRAS.

Survival outcomes were analyzed among the 66 patients who presented with de novo stage IV disease at diagnosis (to minimize confounding by stage). At the last follow-up, 44% of patients with metastatic CRC had died. The median overall survival (OS) for the Stage IV cohort was 43.6 months (95% confidence interval [CI]: 28.7—not reached, Figure 2). KRAS mutation status was significantly associated with poorer OS. On univariate Cox regression, patients with KRAS-mutant tumors had over a three-fold higher risk of death (hazard ratio [HR] 3.52, 95% CI 1.59–7.76; *p* = 0.002, Table 3) compared to those with KRAS wild-type tumors (Figure 3) and this remained significant in multivariate Cox regression analysis (Table 4). In contrast, no significant differences in OS were observed based on patient age (*p* = 0.47), sex (*p* = 0.65), primary tumor sidedness (left vs. right, *p* = 0.18), BRAF mutation status (*p* = 0.22), or MSI status (*p* = 0.30) (Supplementary Figures S1–S4) likely due to limited sample size.

**Table 4:** Multivariable Cox regression analysis.

Characteristic	HR ^1^	95% CI ^1^	*p*-Value
Age at analysis/date at diagnosis	1.00	0.92, 1.08	>0.9
Gender			
Female	—	—	
Male	1.53	0.62, 3.78	0.4
Location			
DC/sigmoid	—	—	
AC	1.05	0.33, 3.32	>0.9
Rectum	0.74	0.29, 1.88	0.5
TC	0.99	0.21, 4.69	>0.9
KRAS			
Absent	—	—	
**Present**	**4.53**	**1.63, 12.6**	**0.004**
MS			
Stable	—	—	
Equivical	3.18	0.62, 16.2	0.2
High	0.46	0.06, 3.61	0.5

^1^ HR = hazard ratio, CI = confidence interval.

## 4. Discussion

YO-CRC represents a molecularly distinct subgroup of the colorectal cancer population with increasing incidence in Western countries. In our study, we identified clinical and prognostic heterogeneity of young-onset colorectal cancer with distinct patterns of molecular alterations. We identified an overall lower rate of KRAS mutation, while BRAF V600E mutations were noted to be present in only 3.6% of patients. While KRAS mutation emerged as the strongest adverse prognostic marker, the presence of targetable alterations such as HER2 amplification, POLE mutations, and MSI-H status in a subset of patients points to actionable avenues for personalized treatment. Notably, the lack of survival difference based on tumor sidedness or BRAF mutation contrasts with patterns seen in older populations, suggesting distinct disease biology in younger patients. Notably, although KRAS mutations were relatively at lower rates, the vast majority of KRAS mutations were exon-2 mutations, including patients who presented with non-metastatic disease at the time of diagnosis.

The molecular underpinnings of YO-CRC are not well defined, with highly varied data reporting distinct results and survival outcomes. For example, an original study suggested an increased incidence of KRAS mutation among patients with YO-CRC [17]. In this study, investigators reported a 54% rate of KRAS mutations, which is above the average KRAS mutation rate of 38–45% seen in the overall CRC population [18]. Another study also suggested a higher incidence of KRAS mutations among young adults with colorectal cancer, with an incidence of 50.5% [19]. Some studies suggest a lower incidence of KRAS mutation but higher rates of exon 2 mutations among young adults [20,21,22]. Our results align with these findings, as we observed a lower incidence of KRAS mutations with a predominant pattern of exon-2 mutations, indicating a distinct pattern of KRAS mutations in younger populations. These differences in incidence of KRAS mutations among young adults can potentially be explained by heterogeneity of study populations with distinct epidemiological risk factors and the impact of environmental factors on driver oncogenic alterations. Another potential explanation could be sampling biases in different academic institutions where patients from rural areas are likely to be included. In our study, we included patients from an institutional network, which includes the underserved population who live in rural areas.

A recent study from Australia suggested a significantly higher incidence of BRAF V600E mutations among patients with colorectal cancer and age 40 or below [23]. It is important to note that so far, the majority of the US-based cohort studies reported otherwise, with decreased incidences of BRAF mutations among the younger population [24,25]. In our study, we also identified a relatively lower incidence of BRAF mutations among patients with YO-CRC, including class I BRAF mutations, which is consistent with the reported evidence from US-based databases. Our data provides further evidence that the incidence of BRAF mutations is less common in YO-CRC compared to patients with adult-onset CRC. HER2 amplification in our study was seen in 4.5% of patients, and this is similar to the overall rate of 3–5% seen in adult-onset CRC [26].

In our study, we also observed high rates of POLE/POLD1 mutations (~10%) among patients with YO-CRC. The incidence of POLE/POLD1 mutations among the overall CRC population has been reported to be 5–6% in various cohort studies, suggesting that patients with YO-CRC may have higher rates of POLE/POLD1 mutations [27,28]. However, notably, some of these patients did not have high-TMB, and only four patients (3.6%) had ultra-high TMB (>30 mutations/Mb), indicating some of these alterations can be seen as passenger mutations in the younger population. In our study, the rate of CNV was 56% which is consistent with the increased rate of CNV seen in MSS CRC [29]. Further studies with larger cohorts are warranted to better define the role and biological characteristics of POLE/POLD1 mutations and CNVs among patients with YO-CRC.

Our study also reveals that KRAS mutations are an independent prognostic factor for patients with YO-CRC, similar to those with adult-onset CRC [30,31,32,33]. Recently, drug discovery research resulted in practice-changing developments in KRAS targeting with novel covalent molecules that inhibit KRAS G12C oncoprotein [33,34,35]. Notably, further research is ongoing with allele-specific inhibitors such as KRAS G12D as well as panKRAS and panRAS inhibitors [18,32,36,37]. Our study underscores the importance of KRAS therapeutic development, as future discoveries will likely improve survival outcomes of patients with colorectal cancer, particularly those with YO-CRC. It is, therefore, important to perform molecular profiling early in the course of the disease and identify potentially actionable molecular alterations, including KRAS oncogene and other driver oncogenes, to develop strategies for clinical trial enrollment to improve outcomes of patients with CRC [38,39].

Our study limitations include the retrospective nature of the study, the size of the patient population, inherent limitations of retrospective data collection, and lack of precise follow-up information; therefore, our findings should be interpreted cautiously. A major strength of our study is that it represents a comprehensive molecular analysis of an understudied patient population with rigorous clinical and molecular data and the inclusion of patients from the suburban network of the institution, which represents an understudied population in the United States and Western Countries. Further prospective studies are warranted to validate our findings and better define the molecular and epidemiological underpinnings of YO-CRC.

## 5. Conclusions

In our study, we identified several distinctions in the genomic profile of patients with YO-CRC, suggesting biological differences in this population. We identified KRAS mutations as independent prognostic biomarkers for which drug development remains a major unmet need. Comprehensive genomic analysis should be integrated into the routine evaluation of YO-CRC to guide risk stratification and therapeutic decision-making, and future studies should explore novel strategies for improving outcomes in KRAS-mutant and other high-risk molecular subgroups.

## Figures and Tables

**Table 1:** Clinical and pathologic characteristics of young-onset colorectal cancer patients (*n* = 110).

Characteristic	Stage I–III (*n* = 44)	Stage IV (*n* = 66)	Total (*n* = 110)
**Age at diagnosis** (median, range)	44 (25–49)	45 (26–49)	44.5 (25–49)
**Gender**			
Female	16 (36%)	24 (36%)	40 (36%)
Male	28 (64%)	42 (64%)	70 (64%)
**Race/Ethnicity**			
White	33 (75%)	59 (89%)	92 (84%)
African American	9 (20%)	3 (4.5%)	12 (11%)
Asian	0 (0%)	2 (3.0%)	2 (1.8%)
American Indian/Alaska Native	1 (2.3%)	0 (0%)	1 (0.9%)
Pacific Islander	0 (0%)	1 (1.5%)	1 (0.9%)
Unreported/Not Disclosed	1 (2.3%)	1 (1.5%)	2 (1.8%)
**Primary Tumor Location**			
Descending/Sigmoid Colon	18 (41%)	30 (45%)	48 (44%)
Rectum	16 (36%)	20 (30%)	36 (33%)
Ascending Colon	6 (14%)	10 (15%)	16 (15%)
Transverse Colon	4 (9.1%)	6 (9.1%)	10 (9.1%)
**Stage at Diagnosis**			
Stage I	6 (14%)	0 (0%)	6 (5.5%)
Stage II	3 (6.8%)	0 (0%)	3 (2.7%)
Stage III	13 (30%)	0 (0%)	13 (12%)
Stage IV	22 (50%)	66 (100%)	88 (80%)

**Table 2:** Molecular and genomic characteristics of young-onset colorectal cancer (*n* = 110).

Molecular Characteristic	Stage I–III (*n* = 44)	Stage IV (*n* = 66)	Total (*n* = 110)
**KRAS mutation status**	–	–	–
Wild-type (no mutation)	30 (68%)	40 (61%)	70 (64%)
Mutant	14 (32%)	26 (39%)	40 (36%)
**KRAS mutation codon** (among KRAS-mutant)			
Exon 2 (codon 12/13)	13 (93%)	22 (84%)	35 (88%)
Non-exon 2 (codon 61/146)	1 (7.1%)	4 (17%)	5 (13%)
**NRAS mutation status**			
Wild-type	44 (100%)	65 (98%)	109 (99%)
Mutant	0 (0%)	1 (1.5%)	1 (0.9%)
**BRAF mutation status**			
V600E mutant	2 (4.5%)	2 (3.0%)	4 (3.6%)
Non-V600E mutant	1 (2.3%)	1 (1.5%)	2 (1.8%)
Wild-type	41 (93%)	63 (95%)	104 (95%)
**POLE/POLD1 mutation** (pathogenic)			
Mutant	1 (2.3%)	10 (15%)	11 (10%)
Wild-type	43 (97.7%)	56 (85%)	99 (90%)
**ERBB2 (HER2) amplification**			
Positive	2 (4.5%)	3 (4.5%)	5 (4.5%)
Negative	42 (95%)	63 (95%)	105 (95%)
**Microsatellite (MSI) Status**			
MSI-high	4 (9.1%)	3 (4.5%)	7 (6.4%)
MS-stable	39 (89%)	59 (89%)	98 (89%)
Equivocal/indeterminate	1 (2.3%)	4 (6.1%)	5 (4.5%)
**Tumor Mutational Burden**			
<10 mutations/Mb	23 (52%)	40 (61%)	63 (57%)
10–20 mutations/Mb	12 (27%)	20 (30%)	32 (29%)
>20 mutations/Mb	9 (20%)	6 (9.1%)	15 (14%)
**CNV profile** (somatic copy number)			
Gain only	7 (16%)	8 (12%)	15 (14%)
Loss only	11 (25%)	23 (35%)	34 (31%)
Both gain and loss	7 (16%)	6 (9.1%)	13 (12%)
No CNV (N/A)	19 (43%)	29 (44%)	48 (44%)
**Oncogenic Fusions**			
NTRK fusion (ETV6–NTRK3)	0 (0%)	2 (3.0%)	2 (1.8%)

KRAS: Kirsten rat sarcoma virus, NRAS: neuroblastoma RAS viral oncogene homolog, BRAF: v-Raf murine sarcoma viral oncogene homolog B, POLE: DNA polymerase epsilon, POLD1: DNA polymerase delta 1, ERBB2: human epidermal growth factor receptor, CNV: copy number variation.

**Table 3:** Univariable Cox regression analysis.

Characteristic	HR	95% CI	*p*-Value
Age at analysis/date at diagnosis	1.03	0.96–1.11	0.50
Gender **(male vs. female)**	-	-	-
Female	-	--	-
Male	0.87	0.41–1.86	0.70
Tumor location			
**DC/Sigmoid**	-	-	-
**AC**	1.38	0.48–3.93	0.50
**Rectum**	0.81	0.34–1.95	0.60
**TC**	0.94	0.21–4.26	>0.90
KRAS mutation			
Absent			
Present	3.52	1.59–7.76	0.002
MSI status			
**Stable**	-	-	-
**Equivocal**	3.55	0.77–16.3	0.10
**High**	0.52	0.07–3.90	0.50
TMB			
≥10	-	-	-
<10	1.72	0.77–3.83	0.20

## Data Availability

Data is not available due to institutional restrictions.

## Supplementary Materials

The following supporting information can be downloaded at: https://www.mdpi.com/article/10.3390/cancers17172763/s1, Figure S1: Kaplan-Meier overall survival curves stratified by MS status (Stable, Equivocal, High). Figure S2: Kaplan-Meier overall survival curves stratified by tumor mutational burden (TMB). Figure S3: Kaplan-Meier overall survival curves by primary tumor site (DC/Sigmoid, AC, Rectum, TC). Figure S4: Kaplan-Meier overall survival curves stratified by gender.
