# Supplementary material for: A Comprehensive Molecular and Clinical Study of Patients with Young-Onset Colorectal Cancer
Source: Cancers (Basel). 2025 Aug 25;17(17):2763. doi: 10.3390/cancers17172763 (PMC12427212; doi:10.3390/cancers17172763)

Figure S1: Kaplan-Meier overall survival curves stratified by MS status (Stable, Equivocal, High)

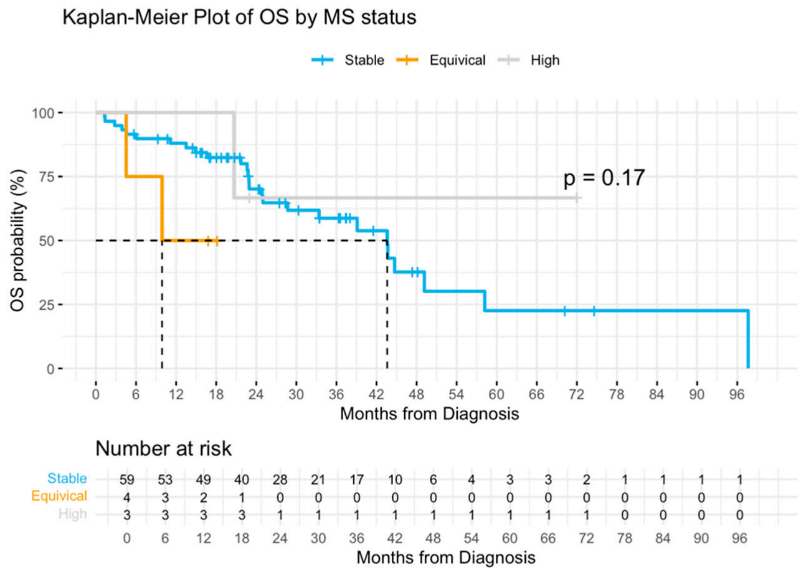


Figure S2 Kaplan-Meier overall survival curves stratified by tumor mutational burden (TMB)

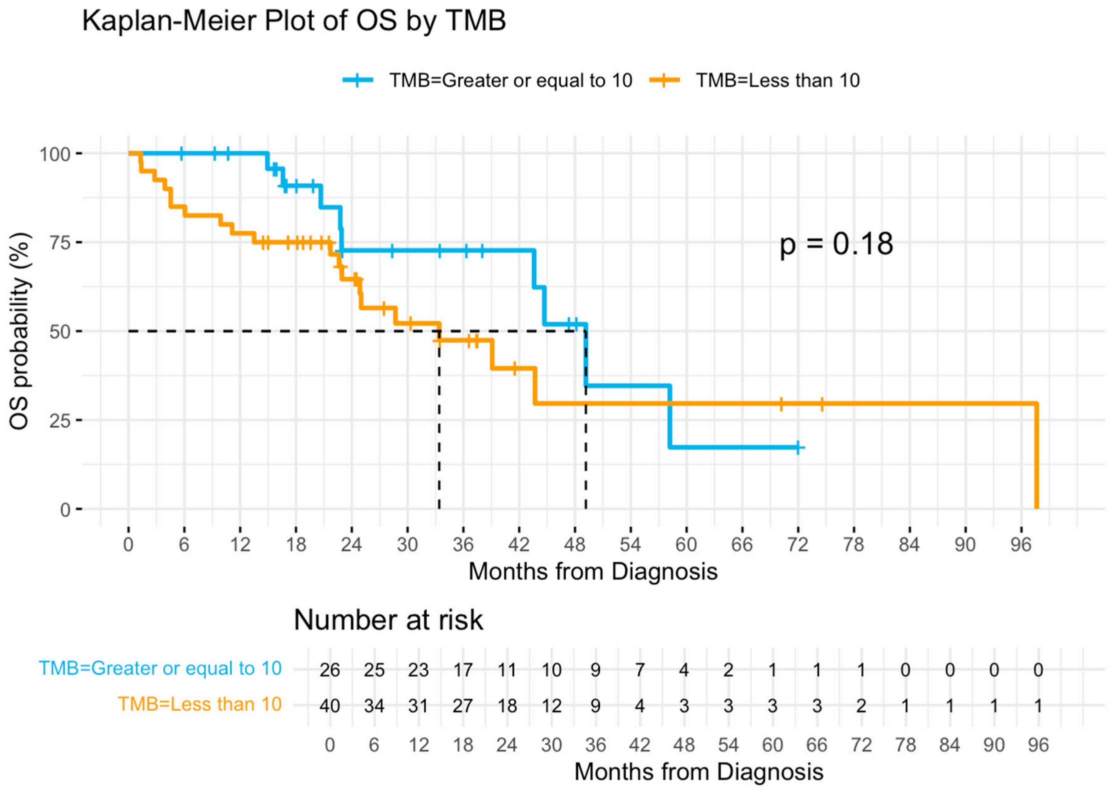


Figure S3: Kaplan-Meier overall survival curves by primary tumor site (DC/Sigmoid, AC, Rectum, TC)

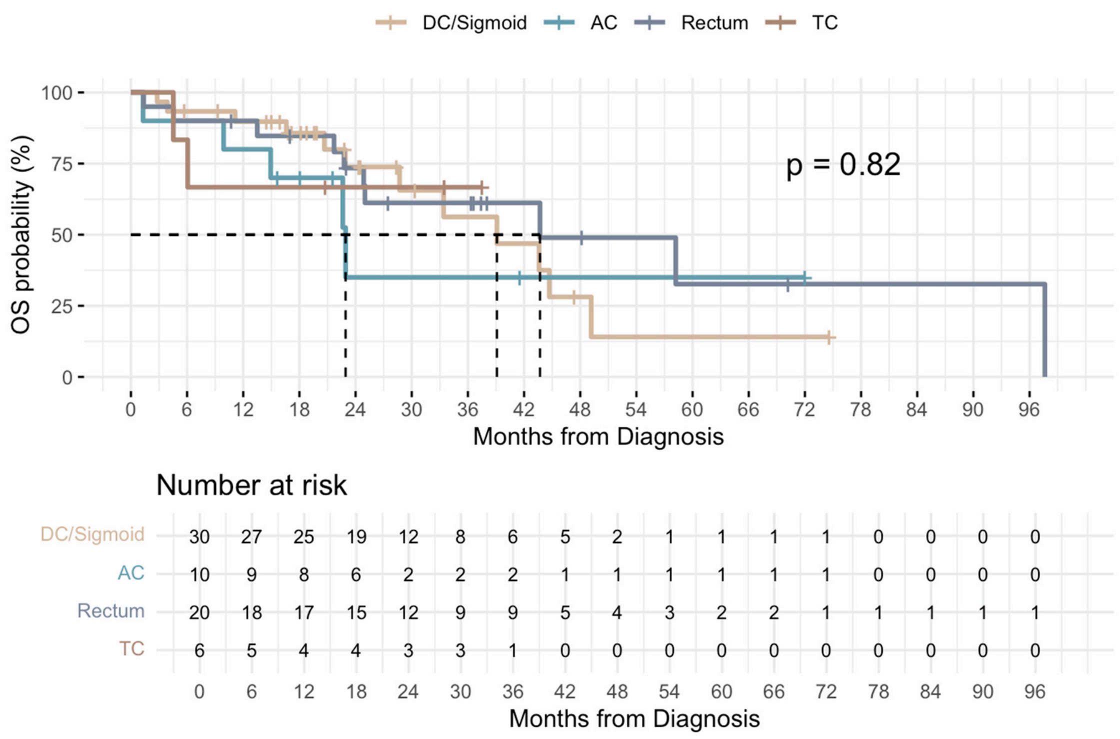


Figure S4: Kaplan-Meier overall survival curves stratified by gender

## Kaplan-Meier Survival Analysis

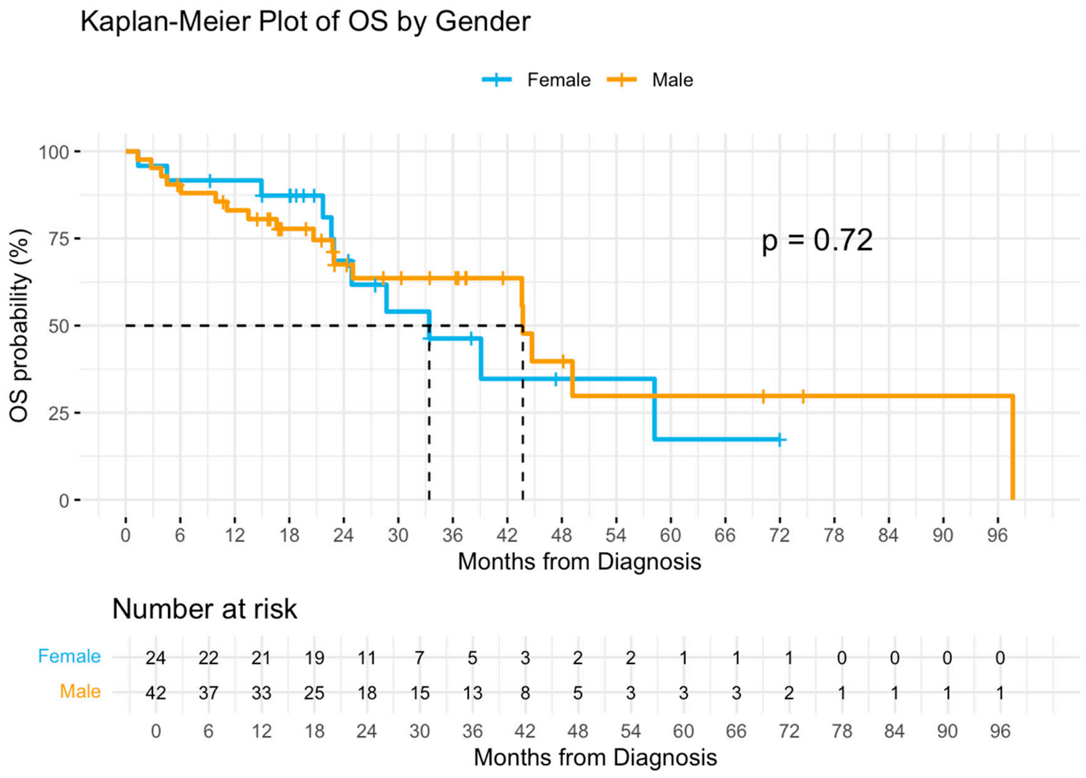

Supplement: Supplementary file 1 [file cancers-17-02763-s001.zip › cancers-3716590-supplementary.pdf]
